# Clinical relevance of guanine-derived urinary biomarkers of oxidative stress, determined by LC-MS/MS

**DOI:** 10.1016/j.redox.2018.11.016

**Published:** 2018-11-26

**Authors:** Ying-Ming Shih, Marcus S. Cooke, Chih-Hong Pan, Mu-Rong Chao, Chiung-Wen Hu

**Affiliations:** a Department of Public Health, Chung Shan Medical University, Taichung 402, Taiwan; b Division of Chest Medicine, Department of Internal Medicine, Changhua Christian Hospital, Changhua 500, Taiwan; c Oxidative Stress Group, Department of Environmental Health Sciences, and Biomolecular Sciences Institute, Florida International University, Miami, FL 33199, USA; d Institute of Labor, Occupational Safety and Health, Ministry of Labor, New Taipei City 221, Taiwan; e Department of Occupational Safety and Health, Chung Shan Medical University, Taichung 402, Taiwan; f Department of Family and Community Medicine, Chung Shan Medical University Hospital, Taichung 402, Taiwan

**Keywords:** 8-oxodGuo, 8-oxo-7,8-dihydro-2′-deoxyguanosine, 8-oxoGua, 8-oxo-7,8-dihydroguanine, 8-oxoGuo, 8-oxo-7,8-dihydroguanosine, AUC, area under the curve, BER, base excision repair, COPD, chronic obstructive pulmonary disease, crt, creatinine, dGuo, 2′-deoxyguanosine, DNPH, 2,4-dinitrophenylhydrazine, Gua, guanine, Guo, guanosine, ICUs, intensive care units, LODs, limits of detection, LOQs, limits of quantification, MV, mechanical ventilation, MDA, malondialdehyde, NER, nucleotide excision repair, RCC, respiratory care center, ROC, receiver operating characteristic, ROS, reactive oxygen species, RT, room temperature, Nucleic acid oxidation, Lipid peroxidation, Urine precipitates, COPD, Mechanical ventilation, LC-MS/MS

## Abstract

A reliable and fast liquid chromatography-tandem mass spectrometry method has been developed for the simultaneous determination of three oxidized nucleic acid damage products in urine, 8-oxoguanine (8-oxoGua), 8-oxo-7,8-dihydro-2′-deoxyguanosine (8-oxodGuo) and 8-oxo-7,8-dihydroguanosine (8-oxoGuo). We applied this method to assess the effect of various urine workup procedures on the urinary concentrations of the oxidized nucleic acid products. Our results showed that frozen urine samples must be warmed (i.e., to 37 °C) to re-dissolve any precipitates prior to analysis. We showed that common workup procedures, such as thawing at room temperature or dilution with deionized water, are not capable of releasing fully the oxidized nucleic acid products from the precipitates, and result in significant underestimation (up to ~ 100% for 8-oxoGua, ~ 86% for both 8-oxodGuo and 8-oxoGuo).

With this method, we further assessed and compared the ability of the three oxidized nucleic acid products, as well as malondialdehyde (MDA, a product of lipid peroxidation), to biomonitor oxidative stress in vivo. We measured a total of 315 urine samples from subjects with burdens of oxidative stress from low to high, including healthy subjects, patients with chronic obstructive pulmonary disease (COPD), and patients on mechanical ventilation (MV). The results showed that both the MV and COPD patients had significantly higher urinary levels of 8-oxoGua, 8-oxodGuo, and 8-oxoGuo (P < 0.001), but lower MDA levels, compared to healthy controls. Receiver operating characteristic curve analysis revealed that urinary 8-oxoGuo is the most sensitive biomarker for oxidative stress with area under the curve (AUC) of 0.91, followed by 8-oxodGuo (AUC: 0.80) and 8-oxoGua (AUC: 0.76). Interestingly, MDA with AUC of 0.34 failed to discriminate the patients from healthy controls. Emerging evidence suggests a potential clinical utility for the measurement of urinary 8-oxoGuo, and to a lesser extent 8-oxodGuo, which is strongly supported by our findings.

## 1 Introduction

Reactive oxygen species (ROS) have been strongly associated with cellular aging, cancer and other degenerative diseases because of their potential to damage cellular constituents, such as nucleic acids, proteins and lipids [1]. For oxidatively damaged nucleic acids, guanine (Gua) is the main oxidation target, as it has the lowest redox potential of the nucleobases [2]. The primary oxidation product of 2′-deoxyguanosine (dGuo) is 8-oxo-7,8-dihydro-2'-deoxyguanosine (8-oxodGuo), which is known to lead to G to T transversion mutations during DNA replication [2], [3]. Cancer may result when such mutations occur in tumor suppressor genes or oncogenes [4], [5]. Oxidatively damaged DNA can be repaired, and the repair products are released into the bloodstream and consequently appear in the urine without further metabolism [6]. Broadly, the presence of the modified nucleobase 8-oxo-7,8-dihydroguanine (8-oxoGua) and modified 2′-deoxyribonucleoside (8-oxodGuo) in urine are thought to represent the major repair products of oxidatively damaged DNA in vivo, via the base excision repair (BER), and nucleotide excision repair (NER)/mismatch repair pathways [7], respectively; although recently the NER pathway for 8-oxodGuo has been questioned [8]. Another proposed source of urinary 8-oxodGuo is the hydrolysis of 8-oxodGTP during sanitization of the dGTP pool, by enzymes such as MTH1 [9], [10]. Oxidatively generated damage to RNA occurs more frequently than DNA, because RNA is mostly single stranded and more easily accessible to ROS and RNA is in closer proximity to mitochondria, where the majority of ROS are generated [11]. It is suggested that oxidized RNA may cause errors in translation, causes ribosome dysfunction, and lead to the production of abnormal proteins [11], [12]. The primary oxidation product of guanosine (Guo) is 8-oxo-7,8-dihydroguanosine (8-oxoGuo). However, little is known about how cells respond to oxidized RNA. It is suggested that oxidized RNA could be removed from the precursor pool by RNA surveillance mechanisms [13], [14], perhaps analogous to those for the DNA pool, and hence be a source of 8-oxoGuo in urine.

Presently, it is the accepted interpretation that the excretion of the oxidized nucleosides, 8-oxodGuo and 8-oxoGuo into urine represents the rate of oxidation of guanine in the respective nucleic acids and their precursor pools (reviewed by Poulsen et al. [14]). Urinary 8-oxodGuo and 8-oxoGuo are regarded as important biomarkers of oxidative stress, and have been utilized not only as an indicator of oxidatively generated damage to nucleic acids, but also as a risk factor for cancer, and many degenerative diseases. For example, elevated levels of urinary 8-oxodGuo have been detected in smokers [15], workers exposed to carcinogens [16], patients with various types of cancer, cardiovascular, diabetes and Alzheimer’s disease (as reviewed previously [14], [17]). In comparison, the measurement of urinary 8-oxoGuo, is still a relatively recent procedure, but several studies have demonstrated elevated urinary 8-oxoGuo levels in psychiatric disorders (e.g., depression [18] and schizophrenia [19]), and it has also been reported that high RNA oxidation is associated with all-cause and cardiovascular mortality risk in patients with type 2 diabetes [20]. Unlike 8-oxodGuo and 8-oxoGuo, the application of 8-oxoGua measurement in human is relatively limited, most likely because 8-oxoGua standard is not stable during preparation and storage [21]. One significant study by Loft et al [22] reported a positive association between 8-oxoGua excretion and lung cancer risk among former and never-smokers, indicating some clinical potential for this biomarker.

Chronic obstructive pulmonary disease (COPD) is a progressive condition characterized by airflow restriction associated with an abnormal inflammatory response of the lungs to noxious particles and gases, caused primarily by cigarette smoking. It has been suggested that ROS derived from cigarette smoke, and from leukocytes and macrophages involved in the inflammatory process, damage the lungs and contribute to the pathogenesis of COPD [23]. Various biomarkers of oxidative stress have been evaluated, mostly in the blood of COPD patients, including lipid peroxidation products, protein oxidation products, and antioxidant enzyme activity [24]. Among these biomarkers, the lipid peroxidation product, malondialdehyde (MDA) in serum has been one of the most measured biomarkers. Several studies show an increase in serum MDA levels in COPD patients, compared to healthy controls [25], although the reverse findings have been also reported [24]. However, to date, there are no studies in the literature that measured biomarkers of oxidatively damage DNA or RNA in the urine of COPD patients.

Mechanical ventilation (MV) is extremely important in clinical practice, as it save lives of patients with critical respiratory problems due to a variety of conditions (e.g., respiratory diseases, spinal cord injuries, neuromuscular diseases, coma, drug overdoses). However, MV is also known to induce lung injury that can later prevent successful weaning off MV, and may result in atrophy and contractile dysfunction of the diaphragmatic myofibers [26], [27]. Both higher positive pressures (e.g., cyclic mechanical stretch [28]) and inspired oxygen fractions greater than 21% (i.e., hyperoxia [29]) are proposed to cause oxidative stress. Despite this, no work has been performed to investigate a possible association between urinary biomarkers of oxidative stress and MV.

In the present study, we developed a new isotope dilution LC-MS/MS method for the simultaneous quantification of 8-oxoGua, 8-oxodGuo and 8-oxoGuo in urine. Using this method, we identified the potential underestimation of urinary levels of oxidized nucleoside/nucleobase, due to a contribution from urinary sediments, which are often neglected in these analyses. This method was then applied to the study of healthy subjects (including 68 smokers and 60 non-smokers), COPD patients (n = 87), and MV patients in intensive care units (ICUs; n = 100). We compared four biomarkers of oxidative stress (i.e., 8-oxoGua, 8-oxodGuo, 8-oxoGuo and MDA) to evaluate the applicability of these four biomarkers for their use in future clinical studies to monitor the levels of oxidative stress in vivo.

## 2 Materials and methods

### 2.1 Chemicals

Solvents and salts were of analytical grade. Unlabeled 8-oxoGua, 8-oxodGuo and malondialdehyde tetrabutylammonium salt were purchased from Sigma-Aldrich. The internal standard, [^15^N_5_]-8-oxo-7,8-dihydro-2′-deoxyguanosine ([^15^N_5_]-8-oxodGuo), was from Cambridge Isotope Laboratories. 8-Oxo-7,8-dihydroguanosine (8-oxoGuo) and [^13^C,^15^N_2_]-8-oxo-7,8-dihydroguanosine ([^13^C,^15^N_2_]-8-oxoGuo) were from Toronto Research Chemicals. The [^15^N_5_]-8-oxo-7,8-dihydroguanine ([^15^N_5_]-8-oxoGua) was synthesized as described previously [21], as was [^2^H_2_]-malondialdehyde (d_2_-MDA) [30].

### 2.2 Urine samples collection

This study was approved by both the Institutional Review Boards of Changhua Christian Hospital and Chung Shan Medical University Hospital in Taiwan. All the participants were adults. Written, informed consent was obtained from the participants themselves, or the patient's legal representative for the MV patients, prior to enrollment. A total of 128 healthy subjects (including 60 non-smokers and 68 current smokers) were recruited. For patients, a total of 87 COPD patients (including 43 non-smokers/ex-smokers and 44 current smokers) and 100 MV patients were recruited. Urine samples from each subject was collected in three 15 mL polypropylene centrifuge tubes (3 tubes × 10 mL), maintained at 4 °C during sampling, and stored at − 20 °C until analysis. For healthy subjects, urine was collected while they had their healthy examinations and mid-stream urine was collected. For COPD patients, urine was collected during patient clinic visits and mid-stream urine was collected.

The COPD patients were recruited from a chest specialist outpatient department at Changhua Christian Hospital. The chief presenting symptoms in these patients were shortness of breath and dyspnea. Every patient received a pre- and post-bronchodilator spirometry test. The diagnosis of COPD was established according to Global Initiative for Chronic Obstructive Lung Disease criteria: the forced expiratory volume in 1 s (FEV1), divided by forced vital capacity, is less than 70% at post-bronchodilator phase, and post-brochodilator FEV1 increase is less than 200 cc and 12%. Patients with fever and pneumonia were excluded. Individual information including age, gender, body mass index (BMI), and smoking status were also collected.

The MV patients were recruited from respiratory care center (RCC) at Changhua Christian Hospital. The RCC is a part of the system of intensive care units (ICUs). All admitted patients were referred from the upstream ICUs, such as medical ICUs, surgical ICUs, the coronary care unit and neurological ICU. Patient urine was collected from the urine drainage bag on the first day of admission to RCC, under the ventilation setting of either pressure-assisted controlled ventilation or volume-assisted controlled ventilation, fraction of inspired oxygen ≤ 30% and positive end-expiratory pressure ≤ 5 cm H_2_O. Each enrolled patient was reviewed for demographic and clinical data.

### 2.3 Urine sample preparation

The stored urine samples were thawed at 37 °C for 15 min to release possible 8-oxoGua, 8-oxodGuo and 8-oxoGuo from the precipitate. After vortexing, 20 μL of urine was diluted 10 times with a solution containing 5 ng of [^15^N_5_]-8-oxoGua, 0.5 ng of [^15^N_5_]-8-oxodGuo and 0.5 ng [^13^C,^15^N_2_]-8-oxoGuo as internal standards in 5% (v/v) methanol (MeOH)/1 mM ammonium acetate (AA). Urinary creatinine (crt) was also measured for each sample using the HPLC-UV method described by Yang [31].

The primary stock solution of 8-oxoGua was prepared by initially dissolving 8-oxoGua powder in 0.01 N NaOH (100 μg/mL), followed by dilution with 5% (v/v) MeOH to 1 μg/mL, stored at − 20 °C. Because 8-oxoGua can undergo decomposition [21], the 8-oxoGua standard stock solutions were prepared freshly every week. Meanwhile, the 8-oxodGuo and 8-oxoGuo primary standard stock solutions were prepared by individually dissolving 8-oxodGuo and 8-oxoGuo in deionized water to a final concentration 1 μg/mL, and stored at − 20 °C. For establishing the linear calibration curve, the stock solutions of 8-oxoGua (75 ng/mL), and 8-oxodGuo and 8-oxoGuo (each of 15 ng/mL) were mixed in equal volumes and then serially diluted 1:1 with 5% (v/v) MeOH/1 mM AA to yield appropriate working solutions.

### 2.4 Simultaneous analysis of urinary oxidized nucleosides/nucleobase in urine using online SPE LC-MS/MS

As stated above, the prepared urine samples (50 μL) were injected into an online SPE LC-MS/MS instrument. The online SPE consisted of a 10-port two-position switching valve (Valco, Instruments) and a C18 trap column (75 × 2.1 mm i.d., 5 µm; ODS-3, Inertsil) and was controlled by PE-SCIEX control software (Analyst, Applied Biosystems). After the online sample purification, the sample was directly transferred to a Luna PFP(2) column (150 × 2.0 mm i.d., 5 µm; Phenomenex) to separate the analytes. The column-switching operation and LC gradients used during the online purification and analyte separation are provided in Supplementary Data Table S1. The total run time was 17 min. The target analyte was quantified by an API 4000 QTrap hybrid triple quadrupole linear ion trap mass spectrometer (QqQ-MS/MS, Applied Biosystems) equipped with a TurboIonSpray source. The resolution was set to a peak width (FWHM) of 0.7 Th for both quadrupole Q1 and Q3. The instrumental parameters were optimized by infusion experiments with pure standards in positive ionization mode. The MRM transitions, along with their respective declustering potentials (DP), collision exit potentials (CXP), and collision energies (CE), are provided in Supplementary Data Table S2. The needle voltage was 5.5 kV and nitrogen was used as the nebulizing gas; the turbo gas temperature was set to 500 °C.

### 2.5 Urinary analysis of MDA using online SPE LC-MS/MS

Urinary concentrations of MDA in control and patients were measured by an isotope-dilution LC-MS/MS method described previously by Chen et al. [30]. Briefly, the urine samples were thawed, vortexed, and then warmed to 37 °C for 15 min. After centrifugation, the urine was diluted 10 times with a solution containing d_2_-MDA as internal standard and then derivatized with 2,4-dinitrophenylhydrazine (DNPH) at 37 °C for 70 min, protected from light, to yield MDA-DNPH and d_2_-MDA-DNPH.

### 2.6 Urinary analysis of cotinine using online SPE LC-MS/MS

Urinary cotinine was used to validate self-reported smoking status. Urinary concentrations of cotinine in healthy subjects and COPD patients were measured by an isotope-dilution LC-MS/MS method previously described by Hu et al. [32]. Non-smokers were defined as those who had urinary concentration of below 30 ng cotinine/mg creatinine, as defined elsewhere [33].

### 2.7 Effect of urine thaw temperature on concentrations of 8-oxoGua, 8-oxodGuo and 8-oxoGuo

Twenty healthy subjects were randomly selected from the healthy subjects. Of the two 15 mL tubes of urine, per subject which were stored at − 20 °C, one tube was thawed at room temperature (RT) for 30 min, centrifuged, and the supernatant was used for the analysis. The other tube was thawed at 37 °C for 15 min, centrifuged, and the supernatant was used for the analysis. After addition of the stable isotope labeled internal standards, the samples were analyzed by online SPE LC-MS/MS as described above.

### 2.8 Statistical methods

Mean and SD were used to describe the demographic data (i.e., age and BMI) for study subjects. Because the concentrations of urinary biomarkers were not normally distributed, the median and interquartile ranges (IQR) were used to summarize the distributions of urinary cotinine, 8-oxoGua, 8-oxodGuo, 8-oxoGuo and MDA. Mann-Whitney *U* test was used to compare continuous variables among groups. Wilcoxon Signed Rank Test was used for paired comparisons. Spearman correlation coefficients were used to examine possible relationships between the urinary biomarkers measured in this study. The data were analyzed using the SPSS statistical package (SPSS, version 22).

## 3 Results

### 3.1 Simultaneous analysis of urinary 8-oxoGua, 8-oxodGuo and 8-oxoGuo using online SPE LC-MS/MS

**Fig. 1:**
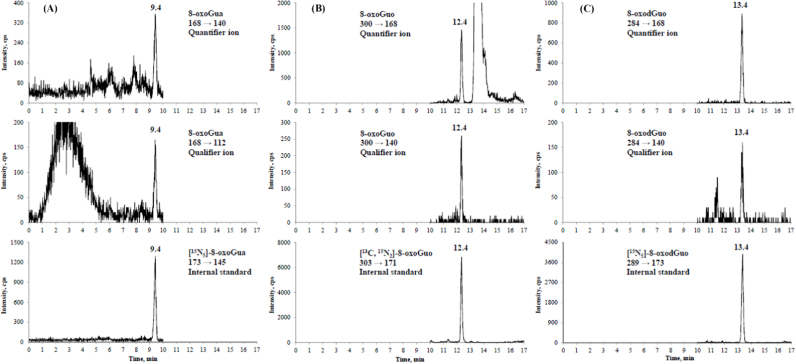
Representative chromatograms of a COPD patient urine spiked with stable isotope labeled internal standards, as measured by LC-MS/MS coupled with online SPE. Multiple reaction monitoring transitions of (A) 8-oxoGua at 9.4 min, (B) 8-oxoGuo at 12.4 min and (C) 8-oxodGuo at 13.4 min.

Fig. 1 represents a typical online SPE LC-MS/MS chromatogram for 8-oxoGua, 8-oxodGuo and 8-oxoGuo in a urine sample spiked with their corresponding internal standards; detailed product ion spectra of 8-oxoGua, 8-oxodGuo and 8-oxoGuo are given in Supplementary Data Fig. S1. The positive ESI mass spectrum of 8-oxoGua (Fig. 1A) contained a [M +H]^+^ precursor ion at *m/z* 168 and product ions at *m/z* 140 (quantifier ion,) and *m/z* 112 (qualifier ion) due to loss of one or two CO groups; a precursor ion at *m/z* 173 and product ion at *m/z* 145 characterized the [^15^N_5_]-8-oxoGua. For 8-oxoGuo (Fig. 1B), the [M +H]^+^ precursor ion was at *m/z* 300 and product ions appeared at *m/z* 168 (quantifier ion) and *m/z* 140 (qualifier ion), resulting from the loss of ribose moiety or together with CO; a precursor ion at *m/z* 303 and product ion at *m/z* 171 characterized the [^13^C,^15^N_2_]-8-oxoGuo. For 8-oxodGuo (Fig. 1C), its [M +H]^+^ precursor ion was at *m/z* 284 and product ions appeared at *m/z* 168 (quantifier ion) and *m/z* 140 (qualifier ion), resulting from the loss of the neutral 2′-deoxyribose moiety or its combination with CO; a precursor ion at *m/z* 289 and product ion at *m/z* 173 characterized the [^15^N_5_]-8-oxodGuo.

With this online SPE LC-MS/MS method, it was interesting to note that the transitions of *m/z* 168 → 140 and 168 → 112 were also detected at the retention times at 12.4 min and 13.4 min, corresponding to 8-oxoGuo and 8-oxodGuo, respectively (Supplementary Fig. S2), suggesting that artefactual 8-oxoGua could be generated, from 8-oxoGuo and 8-oxodGuo, during the ionization process [21]. This finding highlights the importance of an efficient separation of 8-oxoGua from 8-oxodGuo and 8-oxoGuo during chromatography to avoid the overestimation of 8-oxoGua.

The limits of quantification (LOQ) were defined as the lowest concentration in urine that could be reliably and reproducibly measured with values for accuracy, intraday imprecision and inter-day imprecision < 20%. Using the present method, the LOQs in urine were 0.1 ng/mL for 8-oxoGua (5.0 pg on column), 0.007 ng/mL for 8-oxodGuo (0.35 pg on column) and 0.009 ng/mL for 8-oxoGuo (0.45 pg on column). The limits of detection (LODs) in urine, defined as the lowest concentration that gave a signal-to-noise ratio of at least three, were 0.03 ng/mL (1.5 pg) for 8-oxoGua, 0.002 ng/mL (0.1 pg) for 8-oxodGuo and 0.003 ng/mL (0.15 pg) for 8-oxoGuo on column.

Linear calibration curves, covering the entire working range (19.5–5000 pg for 8-oxoGua, and 3.9–1000 pg for both 8-oxodGuo and 8-oxoGuo), were obtained by serial dilution of the calibration standards with deionized water. Each calibrator contained 5 ng of [^15^N_5_]-8-oxoGua, 0.5 ng of [^15^N_5_]-8-oxodGuo and 0.5 ng [^13^C,^15^N_2_]-8-oxoGuo. Linear regression was calculated with non-weighting and non-zero-forced, and the following linear equations and the correlation coefficients (r^2^) were obtained: y = 0.4204x + 0.0049 (r^2^ = 0.9992) for 8-oxoGua, y = 1.5754x - 0.0041 (r^2^ = 0.9998) for 8-oxodGuo and y = 1.2143x −0.0025 (r^2^ = 0.9998) for 8-oxoGuo. Over the entire concentration range of the calibration curves, the mean observed percentage deviation of back-calculated concentrations was between − 10% and + 5.1% for 8-oxoGua, − 4.0% and + 5.0% for 8-oxodGuo and − 2.0% and + 8.0% for 8-oxoGuo, with an imprecision (CV) < 10%. As the analytes are formed endogenously, related compounds with similar polarities, masses and fragmentation patterns could be expected to be present in the urine, and may potentially interfere with the analyte response. Therefore, for each analyte in urine, the peak identity was further confirmed by comparing the peak area ratios (quantifier/qualifier) with those of the calibrators. As an acceptance criterion, ratios in urine samples did not deviate by more than ± 25% from the mean ratios in the calibrators.

The precision was determined by measuring three urine samples with low, medium, and high concentrations and repeatedly measuring the 8-oxoGua, 8-oxodGuo and 8-oxoGuo in these urine samples. As shown in Table 1, the coefficient of variation (CV) of intraday and interday were 0.7–10.4% for 8-oxoGua, 1.4–5.0% for 8-oxodGuo and 2.9–13.7% for 8-oxoGuo.

**Table 1:** Methodological variability of 8-oxoGua, 8-oxodGuo and 8-oxoGuo measurement in human urine.

	Urine	Intra-assay^a^		Inter-assay^a^	
Compounds	sample	Mean ± SD,	CV^b^	Mean ± SD,	CV
		(ng/mL)	(%)	(ng/mL)	(%)
8-oxoGua	1	4.40 ± 0.11	2.4	3.85 ± 0.40	10.4
	2	9.10 ± 0.26	2.9	8.87 ± 0.48	5.5
	3	14.7 ± 0.11	0.7	14.7 ± 0.11	0.7
8-oxodGuo	1	2.37 ± 0.09	3.9	2.21 ± 0.11	5.0
	2	4.07 ± 0.06	1.4	4.06 ± 0.18	4.4
	3	9.70 ± 0.14	1.4	9.52 ± 0.35	3.6
8-oxoGuo	1	3.10 ± 0.42	13.7	3.01 ± 0.22	7.3
	2	6.30 ± 0.50	8.0	5.91 ± 0.17	2.9
	3	11.2 ± 0.50	4.5	11.6 ± 0.35	3.0

a Each urine sample was analyzed six times in the intra-day and inter-day tests.

b CV: coefficient of variation.

The recovery was estimated by the addition of an unlabeled 8-oxoGua, 8-oxodGuo and 8-oxoGuo standard mixture at three different amounts (0.25 ng, 0.5 ng and 1 ng for 8-oxoGua, 0.125, 0.25 and 0.5 ng for 8-oxodGuo and 8-oxoGuo) to the pooled crude urine samples. The recovery was calculated from the increase in the quantified target after the addition of 8-oxoGua, 8-oxodGuo and 8-oxoGuo, divided by the amount that was added. The recoveries were 93–106% for 8-oxoGua, 94–101% for 8-oxodG and 109–117% for 8-oxoGuo.

Matrix effects were calculated from the ratio of the average peak area of the internal standard in the urine samples compared to the aqueous calibration standards. The relative change in peak area of the internal standard was attributed to matrix effects, which reflect both online extraction losses and ion suppression due to the urinary matrix. In this study, the urine matrix effects were 43% for 8-oxoGua, 14% for 8-oxodGuo and 29% for 8-oxoGuo, calculated as (1- internal standard peak area _in urine_/internal standard peak area _in water_) × 100%.

### 3.2 Urinary concentrations of 8-oxoGua, 8-oxodGuo and 8-oxoGuo under different thawing temperature

**Fig. 2:**
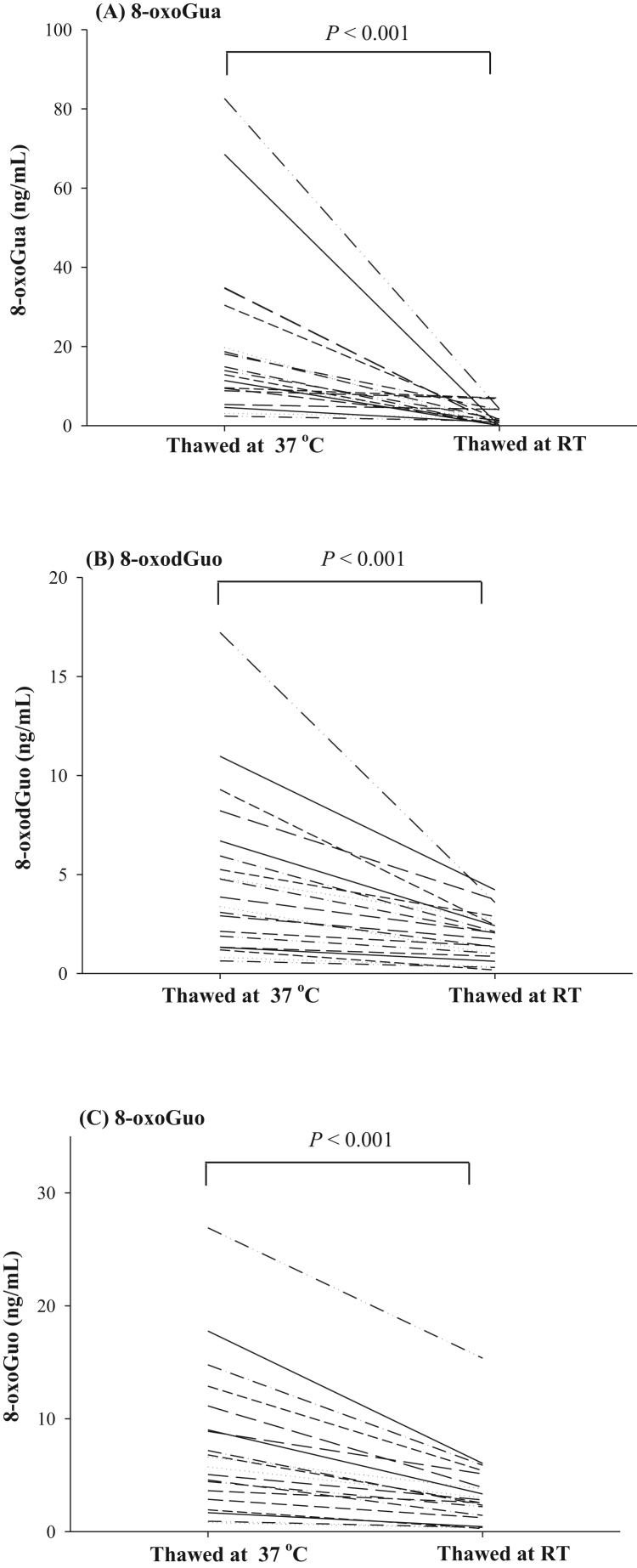
Comparisons of urinary concentrations of (A) 8-oxoGua, (B) 8-oxodGuo, and (C) 8-oxoGuo in urine samples (n = 20 from healthy subjects) following thawing either at RT or 37 °C. The concentration comparison between RT and 37 °C was performed by Wilcoxon Signed Rank Test.

Fig. 2 shows the effect of thawing at RT for 30 min, or at 37 °C for 15 min on urinary 8-oxoGua, 8-oxodGuo and 8-oxoGuo. Levels of 8-oxoGua, 8-oxodGuo and 8-oxoGuo in the urines thawed at RT are significantly lower than those of urines thawed at 37 °C (P < 0.001 for 8-oxoGua; P < 0.001 for 8-oxodGuo; P < 0.001 for 8-oxoGuo, by Wilcoxon Signed Rank Test). The release efficiency (%) from the sediment was calculated from the measured values thawed at RT divided by the measured values thawed at 37 °C. It was estimated that the release efficiency at RT are 0–77% for 8-oxoGua, 14–66% for 8-oxodGuo and from 14% to 71% for 8-oxoGuo as noted in Supplementary Table S3.

### 3.3 Urinary excretion of oxidized guanine lesions and MDA in controls, COPD patients and MV patients

**Fig. 3:**
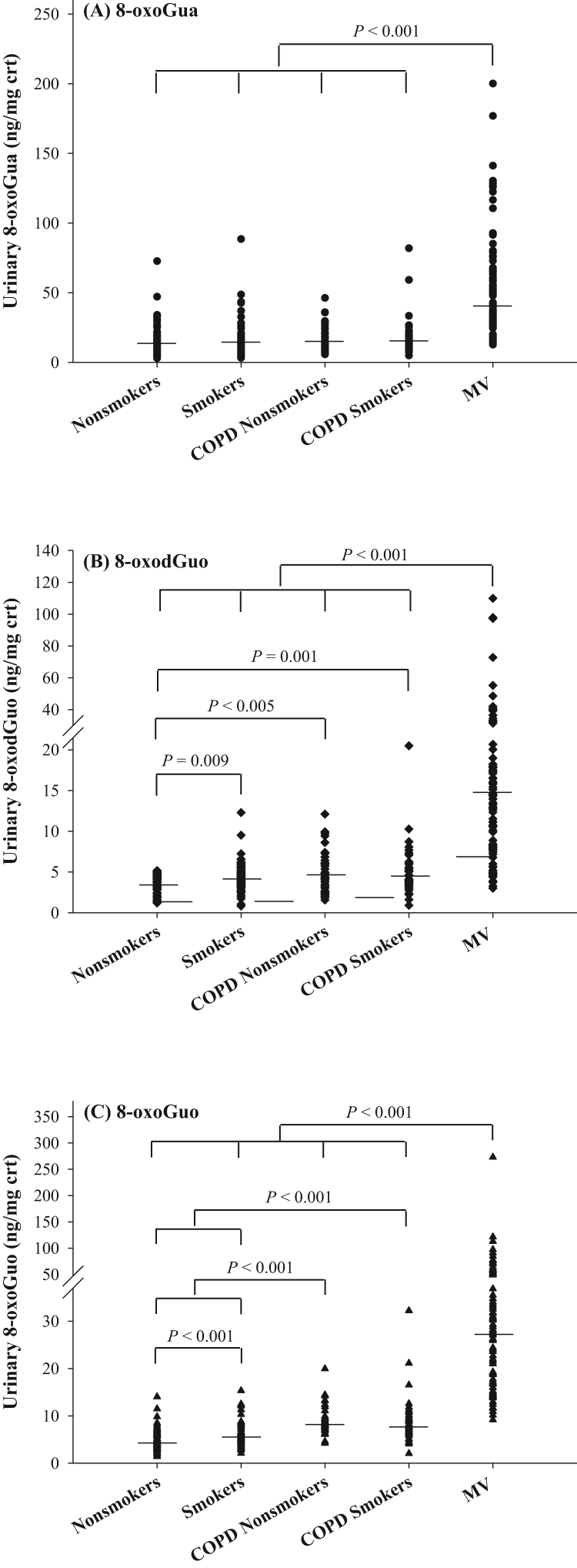
Distribution of (A) 8-oxoGua, (B) 8-oxodGuo, and (C) 8-oxoGuo concentrations in healthy controls (n = 60 non-smokers and 68 smokers), COPD patients (n = 43 non-smokers and 44 smokers), and MV patients (n = 100). Each point represents an individual subject and the horizontal lines represent median values. The comparison was performed by Mann-Whitney *U* test.

The characteristics of patients and controls are presented in Table 2. Both MV patients and COPD patients had higher age than controls (including smokers and non-smokers). The smoking status of control and patients with COPD was validated using urinary cotinine level, and non-smokers were found to have cotinine levels less than 5 ng/mg crt. MV patients had significantly higher urinary excretion of 8-oxoGua, 8-oxodGuo and 8-oxoGuo compared to the COPD patients and the control groups (i.e., control smokers/non-smokers) (P < 0.001), as shown in Fig. 3. For patients with COPD, there was no difference between COPD patient smokers and COPD patient non-smokers for urinary excretion of nucleic acid oxidation markers. However, both COPD patient smokers or COPD patient non-smokers showed increased urinary levels in 8-oxodGuo and 8-oxoGuo (P < 0.005 or P < 0.001) compared to the control non-smokers and/or smokers. Control smokers also showed higher urinary levels of 8-oxodGuo (P = 0.009) and 8-oxoGuo (P < 0.001) than control non-smokers. Interestingly, urinary excretion of 8-oxoGua did not differ between COPD patients, smokers and non-smokers. Similar results were noted when the urinary concentrations were not corrected for creatinine (Supplementary Fig. S3). In contrast to the nucleic acid oxidation markers, MDA did not show higher levels in MV patients or COPD patients than in control (Supplementary Fig. S4).

**Table 2:** Characteristics of the study participants.

Variables	Control		COPD patients		MV patients
	Non-smokers (n = 60)	Smokers (n = 68)	non-smokers/ex-smokers (n = 43)	Smokers (n = 44)	(n = 100)
Age, y	56.7 ± 4^a^	55.5 ± 10	72.5 ± 7.4	64.0 ± 14	69.4 ± 15
BMI, kg/m^2^	25.1 ± 2.4^a^	25.0 ± 3.7	24.1 ± 4.2	22.8 ± 3.9	23.2 ± 4.3
Cotinine, ng/mg crt	1.7 (1.0–2.5)^b^	1338 (602–1916)	1.5 (0.7–2.7)	1050 (380–1554)	NM^c^
ng/mL	1.6 (0.9–2.2)^b^	1163 (646–1728)	1.3 (0.7–2.8)	952 (515–1551)	
8-OxoGua, ng/mg crt	13.1 (8.6–20.2)	14.0 (10.2–18.2)	14.5 (11.6–23.9)	14.8 (11–19.2)	40.2 (28.2–64.8)
ng/mL	11.9 (7.1–18.7)	13.4 (6.6–20.5)	19 (10–27)	17.4 (9.7–23.3)	19 (13.3–30)
8-OxodGuo, ng/mg crt	3.7 (2.8–4.4)	4.1 (3.2–5.2)	4.4 (3.0–6.1)	4.3 (3.5–6.1)	14.6 (8.6–23.1)
ng/mL	3.5 (1.9–5.0)	4.0 (2.4–5.8)	4.4 (2.6–9.9)	4.8 (2.4–6.6)	7.7 (4.7–12)
8-OxoGuo, ng/mg crt	4.3 (3.3–6.2)	5.5 (4.6–7.1)	8.1 (7.2–9.9)	7.9 (6.2–10)	27.6 (16.9–39.8)
ng/mL	4.2 (2.7–6.4)	5.2 (3.4–8.2)	11.2 (5.2–15.3)	7 (5–12.7)	12.9 (8.8–19.2)
MDA, ng/mg crt	125 (85.6–176)	121 (80–197)	87 (61–122)	82 (62–138)	88 (58–129)
ng/mL	122 (66–184)	132 (64–225)	110 (66–148)	84 (52–152)	43 (31–63)

a Mean ± SD.

b Median (IQR).

c NM: not measured.

**Fig. 4:**
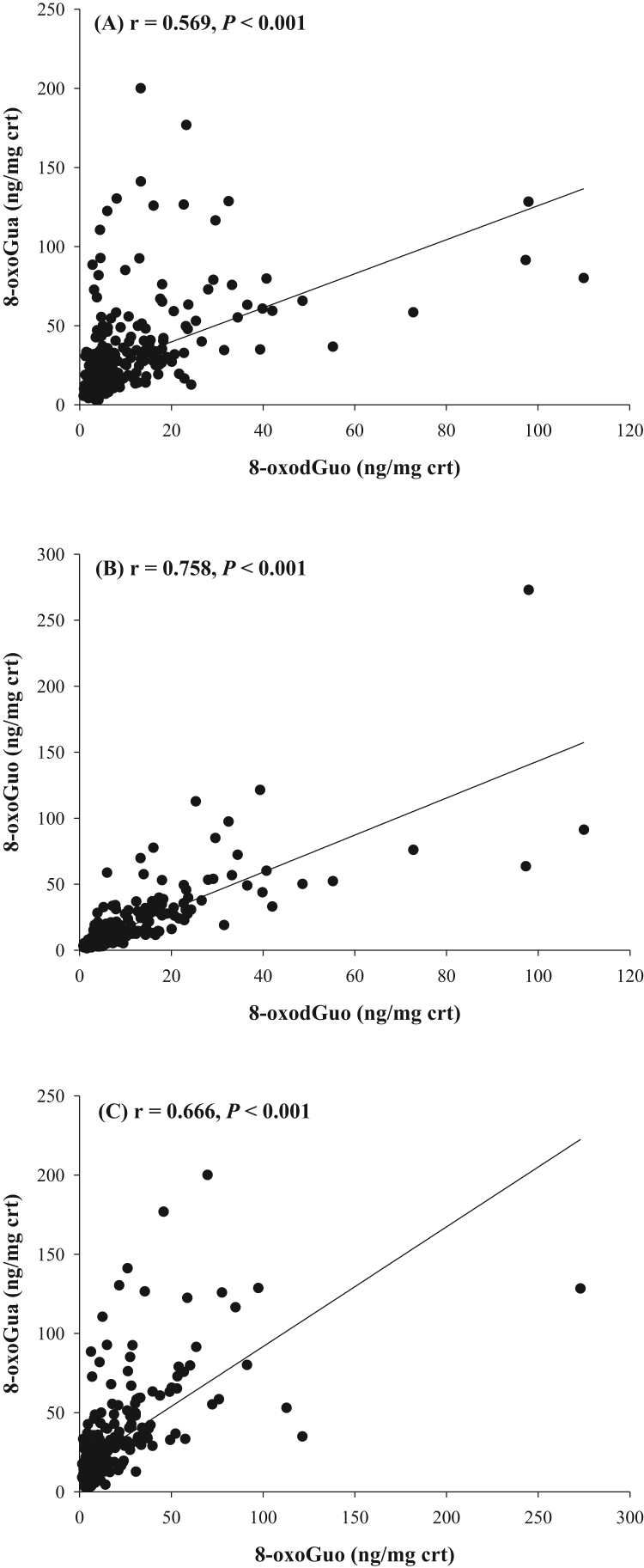
Correlations between the three urinary nucleic acid-derived biomarker concentrations: (A) 8-oxodGuo vs. 8-oxoGua, (B) 8-oxodGuo vs. 8-oxoGuo, and (C) 8-oxoGuo vs. 8-oxoGua. The correlation was estimated by Spearman correlation coefficient.

Possible correlations between 8-oxoGua, 8-oxodGuo and 8-oxoGuo in the urine of control and patients were investigated using Spearman correlation coefficients (Fig. 4). The urinary concentrations of 8-oxodGuo were individually positively associated with urinary 8-oxoGua (*r* = 0.569; P < 0.001) and 8-oxoGuo (*r* = 0.758; P < 0.001). 8-OxoGuo was also positively associated with 8-oxoGua (*r* = 0.666; P < 0.001). Urinary MDA concentrations were not correlated with any of the nucleic acid oxidation markers.

### 3.4 Receiver operating characteristic (ROC) curve for biomarkers of oxidative stress

**Fig. 5:**
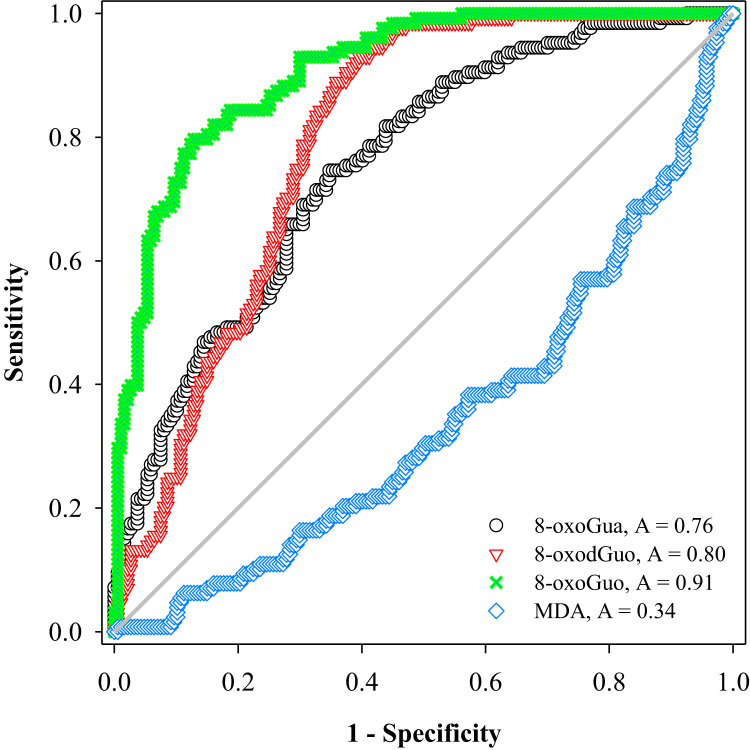
ROC curve analysis of four urinary biomarkers of oxidative stress (measured by LC-MS/MS) for distinguishing patients and healthy subjects. The analysis yielded AUC of 0.91 for 8-oxoGuo, 0.80 for 8-oxodGuo, 0.76 for 8-oxoGua and 0.34 for MDA.

In order to compare the performance of the biomarkers in assessing oxidative stress between healthy controls and patients (including both COPD and MV patients), ROC curve analysis was performed for 8-oxoGua, 8-oxodGuo, 8-oxoGuo and MDA. The results are shown in Fig. 5, and indicated satisfactory results for nucleic acid oxidation markers with area under the curve (AUC) as follows: 0.91 for 8-oxoGuo, 0.80 for 8-oxodGuo and 0.76 for 8-oxoGua. An AUC of > 0.7 [34] for nucleic acid oxidation markers indicated that these markers are considered to be a satisfactory classifier to discriminate patients from healthy controls. In contrast, MDA had an AUC of 0.34, showing no ability to discriminate. A similar trend was found when the urinary biomarkers concentrations were not adjusted for creatinine, but with lower AUCs (Supplementary Fig. S5).

## 4 Discussion

In this study we developed an online SPE LC-MS/MS method for the simultaneous determination of DNA- and RNA-derived oxidatively generated damage products in human urine. Utilization of an online SPE system enables automatic sample preparation, and allows high-throughput urinary analysis for routine applications. In the past decade, several LC-MS based methods have been reported for the measurement of 8-oxodGuo together with 8-oxoGuo in urine [35], [36], [37], [38]. However, it is noted that most methods did not include qualifier ion (except for the work by Henriksen et al. [35]). For low resolution MS (e.g., QqQ-MS/MS), confirmation of analyte identity, using the ratio of the qualifier ion to quantifier ion, is essential for complex biological samples, in which isobaric constituents could be present [39]. Our method includes both quantifier and qualifier ions for each analyte, and possesses a better LODs for all analytes (15 pg for 8-oxoGua, 1.0 pg for 8-oxodGuo and 1.5 pg for 8-oxoGuo) compared to previously reported LC-MS based methods (e.g., 15–50 pg for 8-oxoGua, 4–20 pg for 8-oxodGuo and 3–190 pg for 8-oxoGuo [36], [37], [40], [41]). The high sensitivity of the method will enable automatic detection without manual pretreatment (e.g., pre-concentration by manual SPE) and ensure the availability of essential fragment ions (i.e., qualifier and quantifier ions).

The formation of uncharacterized precipitates frequently occurs in urine samples upon storage at low temperature (e.g., 4, −20 or −80 °C). These precipitates are usually discarded before analysis, although the precipitates could affect the analysis of the analytes of interest [42]. In the literature, only one study has investigated this issue for oxidative stress biomarker analysis, and only for 8-oxodGuo, but the authors note that the 8-oxodGuo can be significantly underestimated, by up to 90%, if the precipitate was not fully re-dissolved [43]. Our results clearly showed that thawing urine at RT for 30 min failed to completely release 8-oxoGua, 8-oxodGuo and 8-oxoGuo from the precipitates, especially for 8-oxoGua. We showed that this led to major underestimations of up to 100% for 8-oxoGua, and up to 86% for both 8-oxodGuo and 8-oxoGuo (Supplementary Table S3). In order to confirm that all three biomarkers can be fully released from the precipitates, by warming the urines at 37 °C, and that this warming step did not lead to artefactual oxidation, we directly compared the concentrations of the three biomarkers in the fresh urines, with those in the urines that had been stored at − 20 °C, and then warmed at 37 °C. The experimental detail and results are provided in Table 3. The results showed that the levels of 8-oxoGua, 8-oxodGuo and 8-oxoGuo in fresh urines are very similar to those in the stored urines following thawing at 37 °C for 15 min, demonstrating nearly 100% release from precipitates was achieved, and with no artefactual oxidation, during warming. The risk of artefactual oxidation could be less in the urine because the molar ratios of Gua:8-oxoGua, dGuo:8-oxodGuo, and Guo:8-oxoGuo are less than 10 [44], unlike in cells the much larger ratios of dGuo:8-oxodGuo (~ 10^6^), or Guo:8-oxoGuo (~ 10^4^–10^6^ [14], [45]).

**Table 3:** Effect of sample workup on 8-oxoGua, 8-oxodGuo and 8-oxoGuo levels in human urine.

	8-oxoGua (ng/mL)				8-oxodGuo (ng/mL)				8-oxoGuo (ng/mL)			
Samples	**Fresh**^a^	**RT**^b^	**RT-5X**^c^	**37 °C**^d^	**Fresh**	**RT**	**RT-5X**	**37 °C**	**Fresh**	**RT**	**RT-5X**	**37 °C**
U1	7.91	5.56	7.10	7.75	1.07	0.67	0.96	0.98	1.93	1.62	1.91	1.91
		(70%)^e^	(90%)	(98%)		(63%)	(90%)	(92%)		(84%)	(99%)	(99%)
U2	18.2	6.18	17.4	18.1	7.54	6.35	7.49	7.51	7.93	6.19	6.93	7.42
		(34%)	(96%)	(100%)		(84%)	(99%)	(100%)		(78%)	(87%)	(94%)
U3	15.4	7.85	11.7	15.1	4.35	4.10	4.52	4.41	4.59	4.16	4.36	4.57
		(51%)	(76%)	(98%)		(94%)	(104%)	(101%)		(91%)	(95%)	(100%)
U4	7.89	2.29	5.40	7.80	2.90	2.96	3.16	3.01	2.86	2.31	2.89	2.84
		(29%)	(68%)	(99%)		(102%)	(109%)	(104%)		(81%)	(101%)	(99%)
U5	3.27	1.08	1.61	2.64	11.5	11.8	12.0	11.6	0.70	0.62	0.67	0.69
		(33%)	(49%)	(81%)		(103%)	(104%)	(101%)		(89%)	(96%)	(99%)
												

a Fresh urine sample was collected into four 2 mL Eppendorf tubes (4 × 1 mL), one tube was measured immediately once collected.

b The remaining three tubes were stored at −20 °C overnight. One tube was thawed at RT for 30 min and centrifuged, and the supernatant was used for analysis (as indicated as RT).

c The frozen urine (1 mL) was thawed at RT for 30 min and then added with 4 mL of deionized water, vortexed and centrifuged, and the supernatant was used for analysis (as indicated as RT-5X).

d The frozen urine was measured following thawing at 37 °C for 15 min.

e Release efficiency: (measured value in the portion of RT (or RT-5X or 37 °C)/measured value in the fresh urine) × 100%.

An alternative approach to releasing the analytes from the precipitates is to encourage them into solution. We showed that diluting the urine with five volumes of deionized water at RT does not fully release oxidized guanine lesions from the precipitates, especially for 8-oxoGua (49–96%, see Table 3). The particularly low release of 8-oxoGua is probably because of a limited solubility of 8-oxoGua in water at neutral pH [21]. In light of our findings, we recommend that thawing at 37 °C is a necessary part of sample preparation for the analysis of oxidized guanine lesions in urine. Previous reports in the literature have used a variety of sample workup steps e.g., thawing of urine at RT [36], removal of precipitates by filtration or centrifugation [38], [46], and diluting the urine with deionized water [47] prior to analysis. We speculate these steps may contribute to some of the apparent assay variability between laboratories, as noted previously [48], [49].

To the best of our knowledge, this is the first study simultaneously demonstrating four different urinary biomarkers of oxidative stress in both patients and healthy subjects. Critically ill patients, such as those on MV, had a high burden of oxidative stress, and showed 3–7 times higher concentrations of oxidized nucleic acids products in urine, compared to the healthy subjects. It was noted that COPD and MV patients had a significantly higher mean age compared to healthy subjects in this study (P < 0.001), which might be related to the higher urinary concentrations of oxidized nucleic acids in the patient group [50]. However, the multiple linear regression analysis revealed that after adjusting for age and BMI, the three biomarker concentrations were highly positively correlated to the subject’s category (healthy subjects or patients) (P < 0.001). This demonstrated that the “disease” was the major factor influencing the urinary concentrations of oxidized nucleic acids. Subsequently, it was surprising that MDA, a widely measured product of lipid peroxidation, did not show significantly higher concentrations in MV or COPD patients compared to healthy subjects, despite the use of robust methodology.

We further compared these four biomarkers of oxidative stress using ROC curve analysis. The results showed that 8-oxoGuo, with the maximum AUC of 0.91, is the biomarker of oxidative stress which best discriminates patients from healthy subjects. Among the three oxidized guanine lesions, 8-oxoGua has the lowest area (AUC: 0.76), suggesting that processes unrelated to the underlying pathology influence urinary 8-oxoGua levels, but the precise reason(s) for this are unclear. One reason might be that diet and gut bacteria may contribute to levels of urinary 8-oxoGua [14], although several studies demonstrate that 8-oxoGua is independent of diet [51], [52].

We combined two biomarkers (8-oxodGuo and 8-oxoGuo) and three biomarkers (8-oxoGua, 8-oxodGuo and 8-oxoGuo) to assess their ability to discriminate between healthy subjects and patients. The results of ROC curve analysis is provided in Supplementary Data Fig. S6. The results showed that 8-oxoGuo alone still gave the best discrimination ability (AUC of 0.91), followed by the two biomarkers (AUC = 0.89) and three biomarkers combinations (AUC = 0.83).

Our results demonstrated that urinary 8-oxoGuo is a more sensitive, non-invasive biomarker of oxidative stress than urinary 8-oxodGuo. Explanation for this might be that RNA is more prone to oxidation than DNA. A previous study showed that the level of RNA damage per nucleoside is 14–25 times greater than that in DNA [53]. As stated earlier, this could be because of its cytosolic location, closer to the mitochondria, its single-stranded structure, and the lack of histone protection. Another factor that may account for the higher susceptibility of RNA to oxidatively generated damage is the iron-binding properties of certain classes of RNA, that can helps catalyze the production of ROS via Haber-Weiss and Fenton reactions [54]. Moreover, it has also been reported that mammalian cells have RNA levels 4.4 times higher than that of DNA by weight [54], representing a quantitatively greater target for ROS. Interestingly, despite RNA being more prone to oxidation, and is present in amounts several times that of DNA, urinary concentrations of 8-oxoGuo are in fact comparable to 8-oxodGuo. This finding led us to suspect that the 8-oxoGuo in the urine may not be the major product of RNA degradation/repair, or both urinary 8-oxoGuo and 8-oxodGuo might mainly reflect the sanitization of precursor pool [55].

It was surprising that urinary MDA, as a biomarker of oxidative stress, failed to discriminate all the patients (COPD and MV patients) from healthy subjects (with an of AUC < 0.5 [34]). When the ROC curve analysis was performed with only healthy controls and MV patients alone (see Supplementary Fig. S7), a similar result was obtained, showing that a low AUC of 0.34 was obtained for MDA, whereas even better AUC was obtained each for 8-oxoGuo (AUC: 1.00), 8-oxodGuo (AUC: 0.95) and 8-oxoGua (AUC: 0.91). In fact, it is interesting that COPD and MV patients showed significantly lower urinary concentrations of MDA than healthy controls, particularly for the MV patients (P < 0.005; Supplementary Fig. S4). As noted above, this could be partially explained by MDA levels being influenced by the diet (e.g., high-fat diet [56]). Previous studies have shown that the animals fed with dietary fat and oils increased lipid peroxidation as measured by elevated blood MDA concentrations [57]. In addition to endogenous lipid peroxidation, the MDA content in food was significantly increased after pan- or deep-frying [58] because of the oxidation of polyunsaturated fatty acids, and the ingested MDA could be taken up and subsequently excreted [59]. Based on the above previous studies, a lower urinary MDA concentration in MV patients may be because MV patients receive a modified diet via enteral feeding, which does not contain fried food, and hence MDA. Previous studies show that blood MDA concentrations are higher in patients with COPD [60] and some other diseases [61]. This discrepancy with our findings could be explained by our use of the robust LC-MS/MS approach, rather than non-specific spectrophotometry, and analysis of different biological matrices (blood vs. urine).

In conclusion, this study describes a simple, rapid, and reliable LC-MS/MS method for the direct determination of urinary 8-oxoGua, 8-oxodGuo and 8-oxoGuo. Application of this method, revealed that the precipitates present in urine are of great importance for the accurate measurement of these biomarkers of oxidative stress. Failure to perform the appropriate sample workup for the precipitates can lead to a severe underestimation of the biomarker levels. We recommend that thawing at 37 °C for 15–20 min (for a 15 mL tube) is necessary for the effective release of maximal levels of 8-oxoGua, 8-oxodGuo and 8-oxoGuo from the precipitates. Our study is also the first to report the analysis of three oxidized guanine-derived products, together with the MDA, in healthy subjects, COPD and MV patients, and to assess their ability to discriminate patients from healthy controls. Surprisingly, 8-oxoGuo (a putative marker of RNA oxidation), rather than the more frequently measured 8-oxodGuo, exhibited a superior ability to discriminate the patients from the healthy subjects, compared to 8-oxodGuo or 8-oxoGua. Conversely, urinary MDA failed to discriminate between the healthy controls and patients. Interestingly, a number of other, recent studies have suggested 8-oxoGuo to have a potential clinical utility [18], [20]. Our findings give further clinical validity to evaluating urinary 8-oxoGuo, although the biological role of 8-oxoGuo in disease remains to be determined, and warrants further investigation.
