# Supplementary material for: Clinical relevance of guanine-derived urinary biomarkers of oxidative stress, determined by LC-MS/MS
Source: Redox Biol. 2018 Nov 26;20:556–65. doi: 10.1016/j.redox.2018.11.016 (PMC6279954; doi:10.1016/j.redox.2018.11.016)
Supplement: Supplementary file 1 — Supplementary material [file mmc1.pdf]

Supplementary Data

Clinical relevance of guanine-derived urinary biomarkers of oxidative stress, determined by LC-MS/MS

Ying-Ming Shih[a,b], Marcus S. Cooke[c], Chih-Hong Pan[d], Mu-Rong Chao[e,*], Chiung-Wen Hu[a,f,*]

[a] Department of Public Health, Chung Shan Medical University, Taichung 402, Taiwan.

[b] Division of Chest Medicine, Department of Internal Medicine, Changhua Christian Hospital, Changhua 500, Taiwan.

[c] Oxidative Stress Group, Department of Environmental Health Sciences, Florida International University, Miami, FL 33199, USA.

[d] Institute of Labor, Occupational Safety and Health, Ministry of Labor, New Taipei City 221, Taiwan

[e] Department of Occupational Safety and Health, Chung Shan Medical University, Taichung 402, Taiwan.

[f] Department of Family and Community Medicine, Chung Shan Medical University Hospital, Taichung 402, Taiwan.

***Corresponding Author:** C.-W. Hu, Department of Public Health, Chung Shan Medical University, Taichung 402, Taiwan. Fax: +886-4-23248179, E-mail: cwhu0823@gmail.com, windyhu@csmu.edu.tw.; M.-R. Chao, Department of Occupational Safety and Health, Chung Shan Medical University, Taichung 402, Taiwan. Fax: +886-4-23248194, Email: chaomurong@gmail.com, mrchao@csmu.edu.tw.

Table S1. Timetable for the column-switching procedure.

Time, min	Eluent I (trap column)		Eluent II (analytical column)		Valve position	Flow rate, ml/min	Remark
	Solvent I[a], %	Solvent II[b], %	Solvent III[c], %	Solvent IV[d], %			
0	100	0	100	0	A	0.2	Injection and washing sample.
2.5	100	0	100	0	B	0.2	Start of elution of 8-oxoGua to the analytical column.
4.4	100	0	100	0	B	0.2	
4.5	70	30	100	0	A	0.2	
4.6	70	30	80	20	A	0.2	
7	70	30	80	20	A	0.2	
7.1	70	30	50	50	A	0.2	
8.2	70	30	50	50	B	0.2	Start of elution of 8-oxoGuo and 8-oxodGuo to the analytical column.
9.5	70	30	50	50	B	0.2	
9.6	0	100	50	50	B	0.2	
10	0	100	50	50	A	0.2	End of elution, trap column cleanup and reconditioning.
12	0	100	50	50	A	0.2	
12.1	100	0	50	50	A	0.2	
13	100	0	0	100	A	0.2	
15	100	0	0	100	A	0.2	
15.1	100	0	100	0	A	0.2	
17	100	0	100	0	A	0.2	

[a]5% (v/v) MeOH containing 1 mM AA; [b]75% (v/v) MeOH containing 1 mM AA; [c]5% (v/v) MeOH containing 5 mM AA; [d]50% (v/v) MeOH containing 1 mM AA.

Table S2. Tandem mass spectrometry parameters for 8-oxoGua, 8-oxodGuo and 8-oxoGuo.

Compound	Q1 mass, amu	Q3 mass, amu	Dwell time, ms	DP[a], V	CXP[b], V	CE[c], eV
8-oxoGua	168	140[d]	200	65	11	25
	168	112[e]	200	65	11	30
[$^{15}N_5$]-8-oxoGua	173	145	200	65	11	25
8-oxodGuo	284	168	100	50	11	20
	284	140	100	50	11	45
[$^{15}N_5$]-8-oxodGuo	289	173	100	50	11	20
8-oxoGuo	300	168	100	45	11	20
	300	140	100	45	11	45
[^{13}C, $^{15}N_2$]-8-oxoGuo	303	171	100	45	11	20

[a]Declustering potential; [b]Collision exit potential; [c]Collision energy; [d]Quantifier ion; [e]Qualifier ion.

Table S3. Urinary concentrations (ng/mL) of 8-oxoGua, 8-oxodGuo and 8-oxoGuo in the urine samples following thawing at RT for 30 min, and 37 ℃ for 15 min.

Urine	8-oxoGua			8-oxodGuo			8-oxoGuo		
	RT	37 ℃	Release efficiency[a]	RT	37 ℃	Release efficiency	RT	37 ℃	Release efficiency
1	0.74	4.61	16%	0.61	1.32	46%	0.44	1.68	26%
2	1.13	11.4	10%	2.75	4.82	57%	3.80	6.61	58%
3	1.48	30.4	5%	2.44	9.29	26%	5.41	12.9	42%
4	4.27	82.6	5%	3.59	17.2	21%	15.4	26.9	57%
5	4.08	5.36	76%	1.73	2.92	59%	2.82	5.07	56%
6	3.96	18.1	22%	2.12	5.94	36%	2.35	7.18	33%
7	6.82	8.81	77%	1.38	2.13	65%	2.57	3.63	71%
8	ND[b]	68.5	0%	4.23	11.0	39%	6.05	17.8	34%
9	ND	19.7	0%	0.96	3.39	28%	3.02	5.74	53%
10	ND	12.9	0%	0.17	1.19	14%	0.27	1.94	14%
11	1.11	2.39	47%	0.30	0.63	48%	0.36	0.92	39%
12	ND	34.8	0%	2.06	3.87	53%	5.12	8.88	58%
13	ND	14.9	0%	1.35	3.09	44%	1.42	4.58	31%
14	1.22	9.43	13%	0.87	1.33	66%	1.23	2.87	43%
15	ND	11.4	0%	2.41	6.70	36%	3.34	9.02	37%
16	1.98	3.10	64%	0.34	0.80	42%	0.31	0.79	39%
17	6.98	9.59	73%	2.89	5.25	55%	2.54	6.80	37%
18	1.65	13.9	12%	1.03	1.89	55%	2.22	4.44	50%
19	ND	34.7	0%	3.74	8.22	45%	3.92	11.1	35%
20	1.26	18.7	7%	2.05	4.78	43%	5.89	14.8	40%

[a]Release efficiency: (measured values thawed at RT/measured values thawed at 37 ℃) × 100%.

[b]ND: not detected.

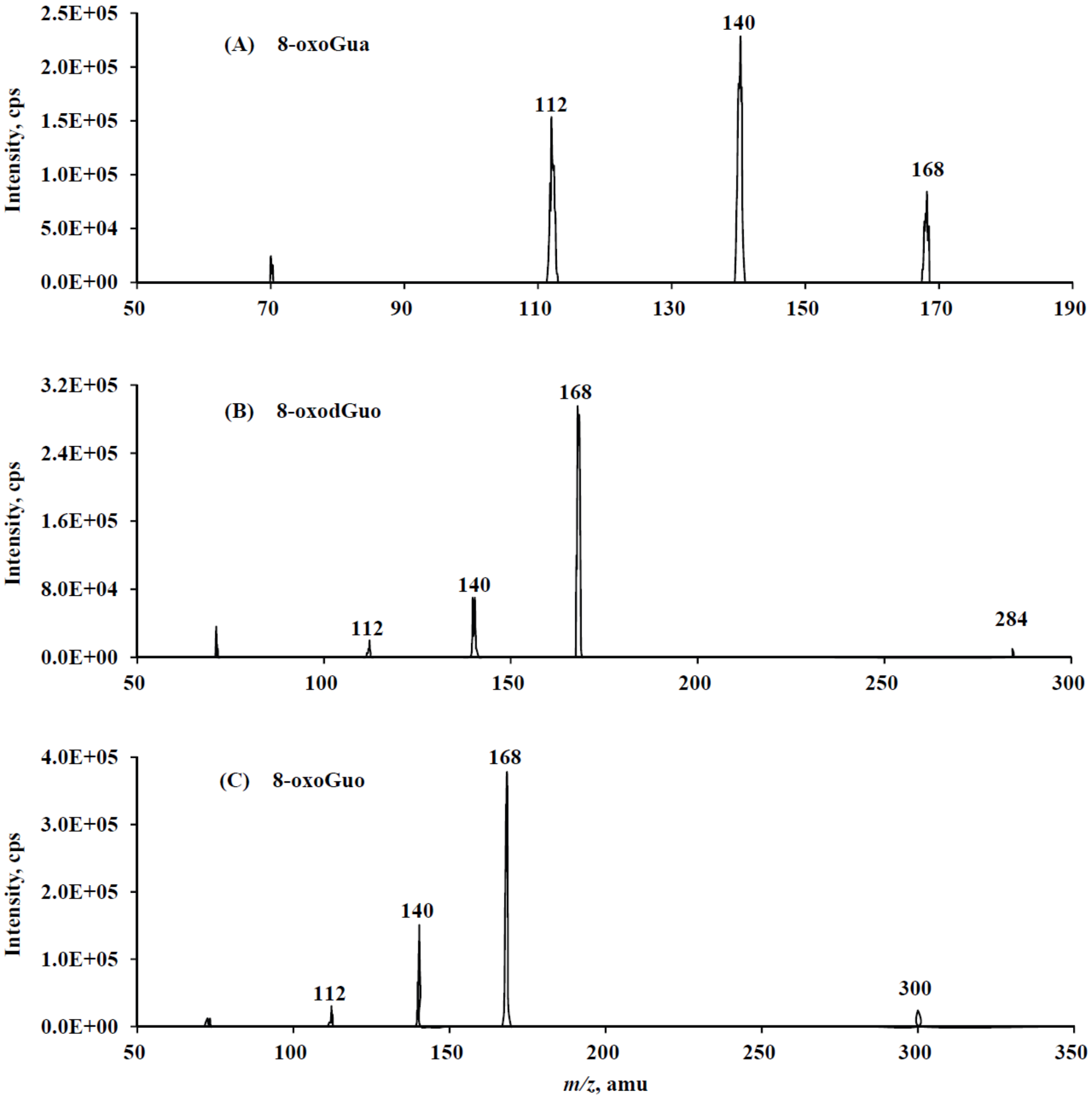

Figure S1. Positive-ion electrospray MS/MS spectra of $[M + H]^+$ of (A) 8-oxoGua, (B) 8-oxodGuo and (C) 8-oxoGuo.

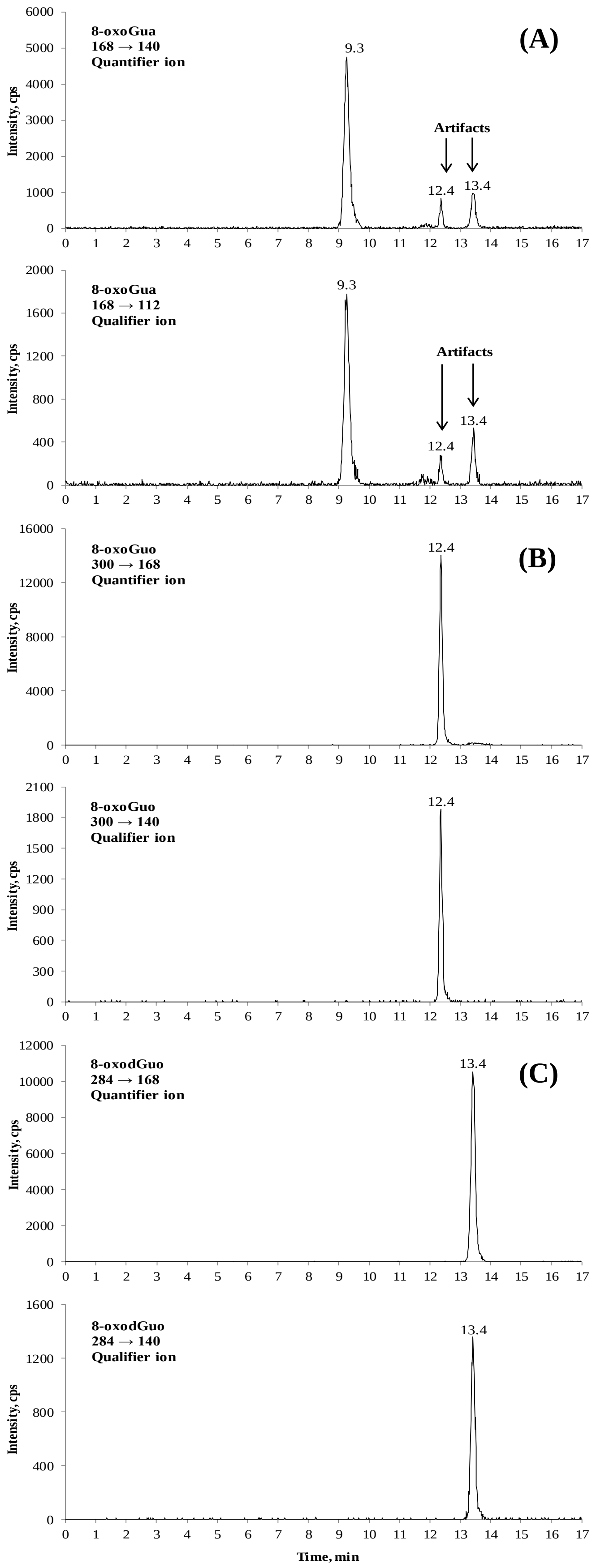

Figure S2. Chromatograms of (A) 8-oxoGua, (B) 8-oxoGuo, and (C) 8-oxodGuo in a urine of an MV patient, as measured by LC-MS/MS coupled with online SPE. Artefactual 8-oxoGua was observed at 12.4 min and 13.4 min, corresponding to 8-oxoGuo and 8-oxodGuo, respectively.

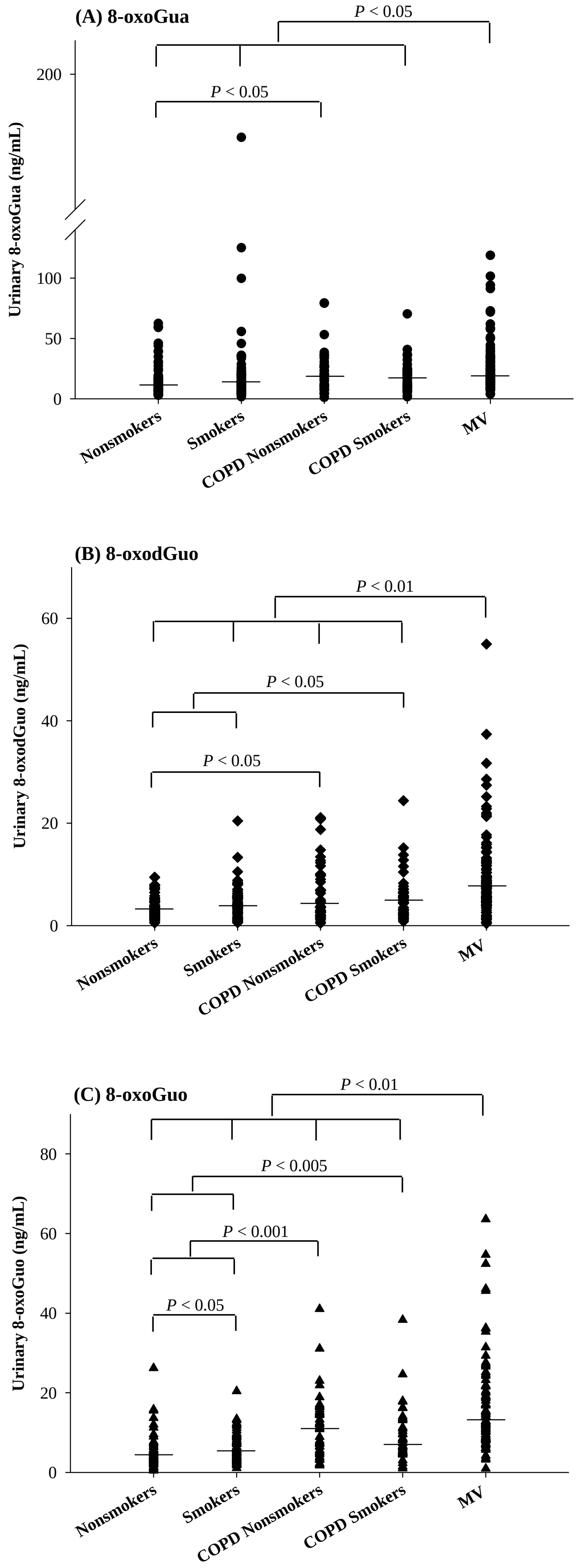

Figure S3. Urinary concentrations of (A) 8-oxoGua, (B) 8-oxodGuo and (C) 8-oxoGuo in control, COPD pateints and MV patients without creatinine adjustment. Each point represents an individual subject and the horizontal lines represent median values. The comparison was performed by Mann-Whitney *U* test.

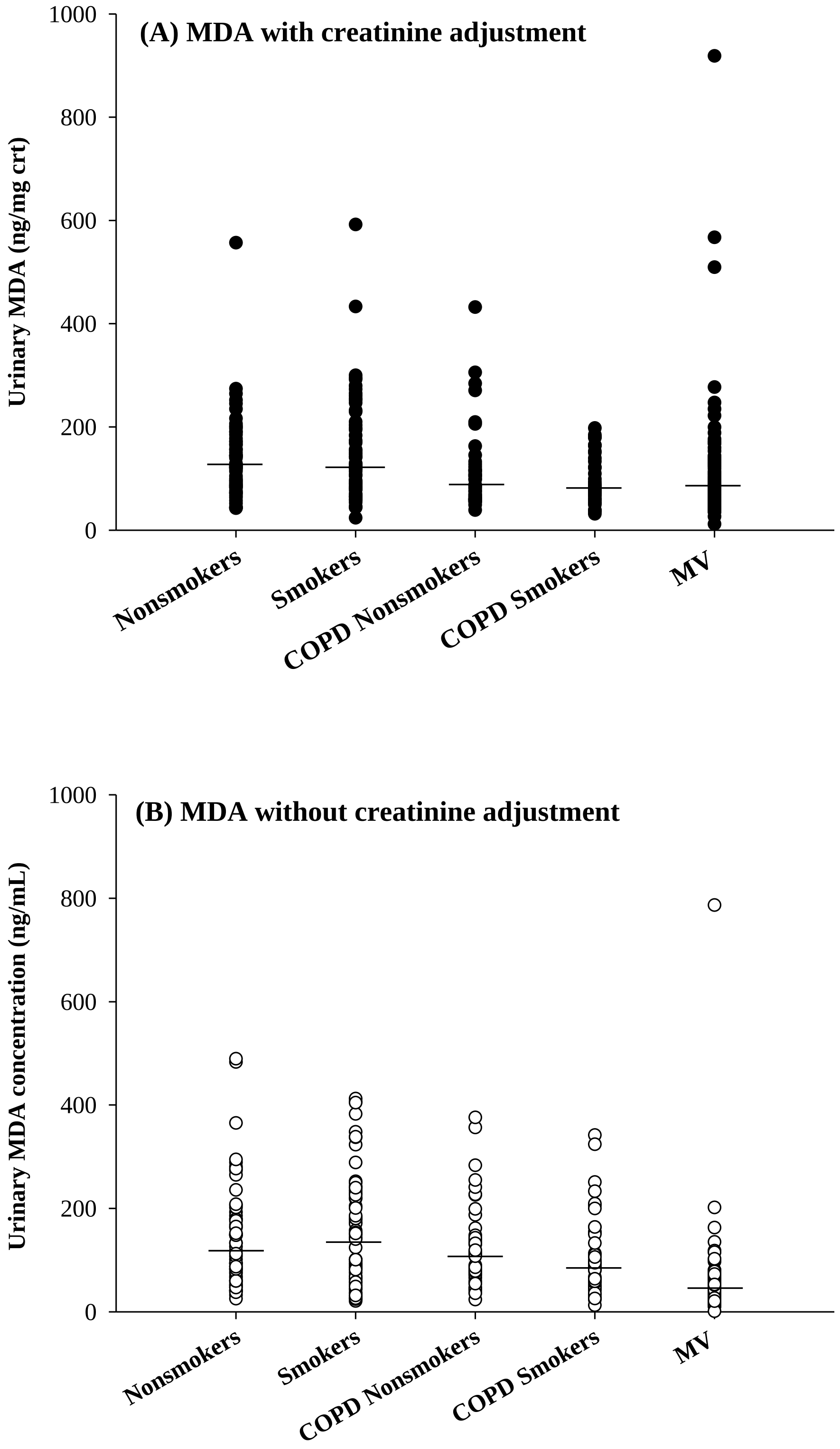

Figure S4. Urinary concentrations of MDA (A) with creatinine adjustment, and (B) without creatinine adjustment in control, COPD pateints and MV patients. Each point represents an individual subject and the horizontal lines represent median values. MV patients had a significantly lower median MDA concentration than healthy controls with or without creatinine adjustement (P < 0.005, by Mann-Whitney *U* test).

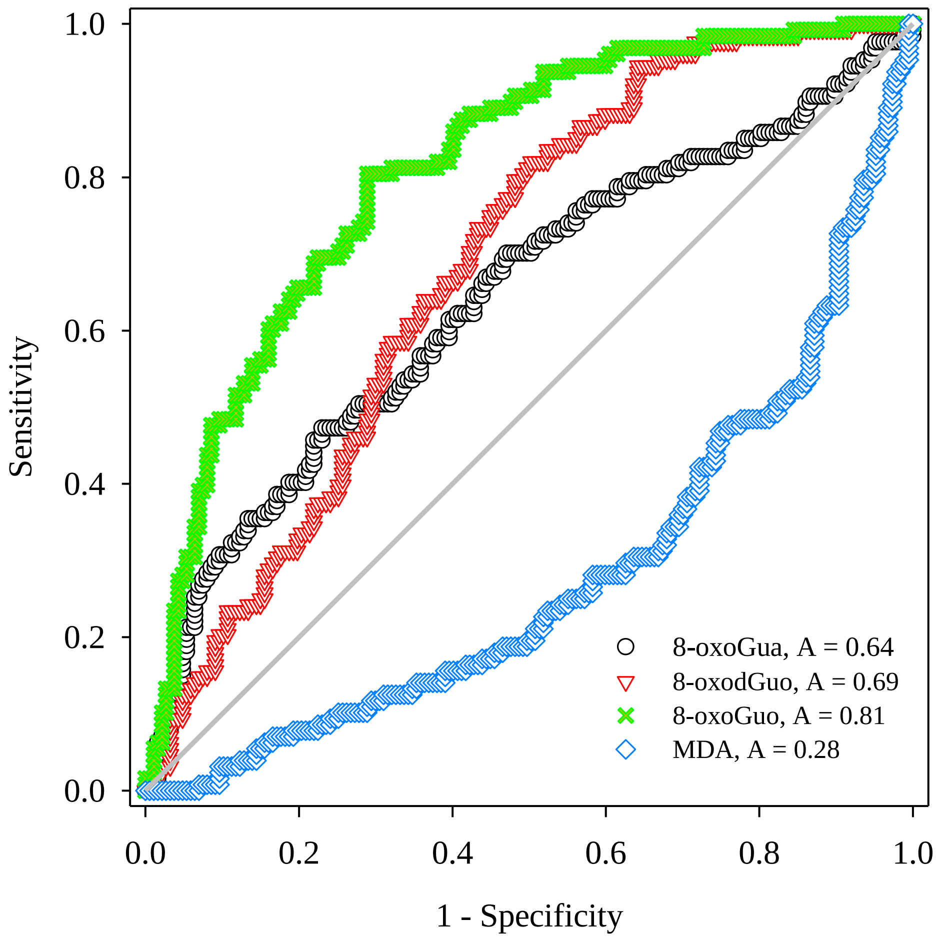

Figure S5. ROC curve analysis of 8-oxoGua, 8-oxodGuo, 8-oxoGuo and MDA. Urinary biomarkers concentrations were not adjusted for creatinine. Urinary 8-oxoGuo yielded the maximum area (A = 0.81) under the curve, followed by 8-oxodGuo (A = 0.69), 8-oxoGua (A = 0.64) and MDA (A = 0.28).

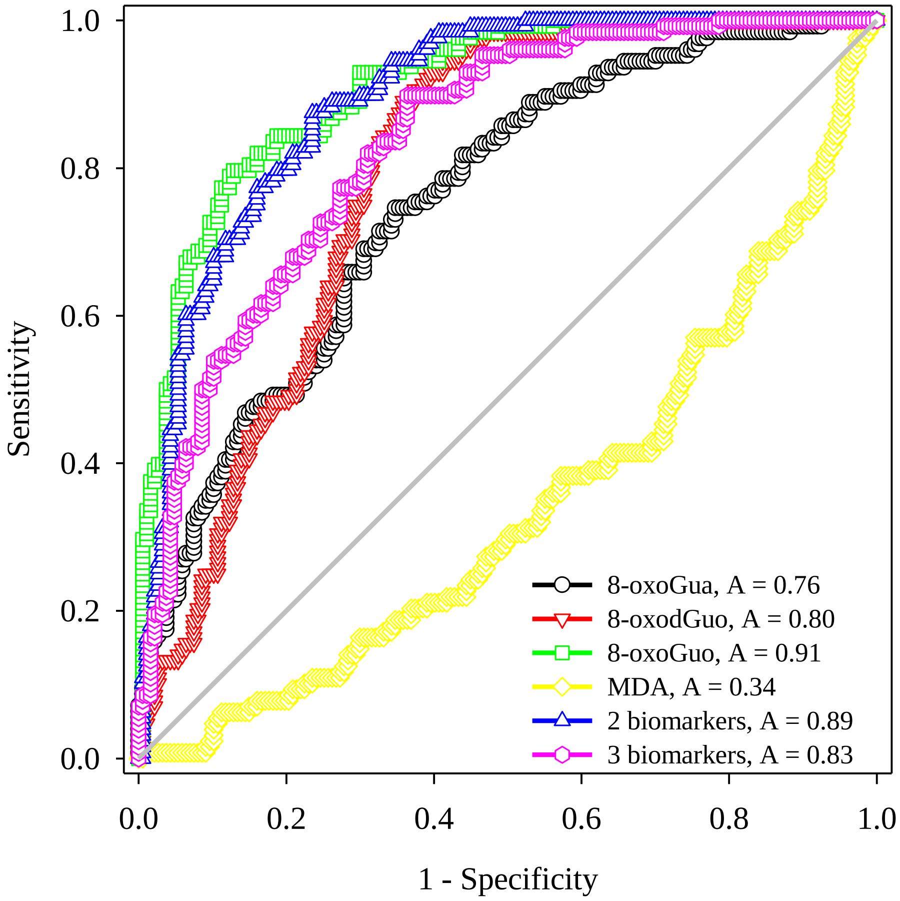

Figure S6. ROC curve analysis of multiple biomarkers. Urinary biomarkers concentrations were adjusted for creatinine. Two biomakers represents the sum of 8-oxodGuo and 8-oxoGuo while three biomarkers represents the sum of 8-oxoGua, 8-oxodGuo and 8-oxoGuo. Urinary concentration 8-oxoGuo yielded the maximum area (A = 0.91) under the curve, followed by two biomarkers (A = 0.89), three biomarkers (A = 0.83), 8-oxodGuo (A = 0.80), 8-oxoGua (A = 0.76) and MDA (A = 0.34).

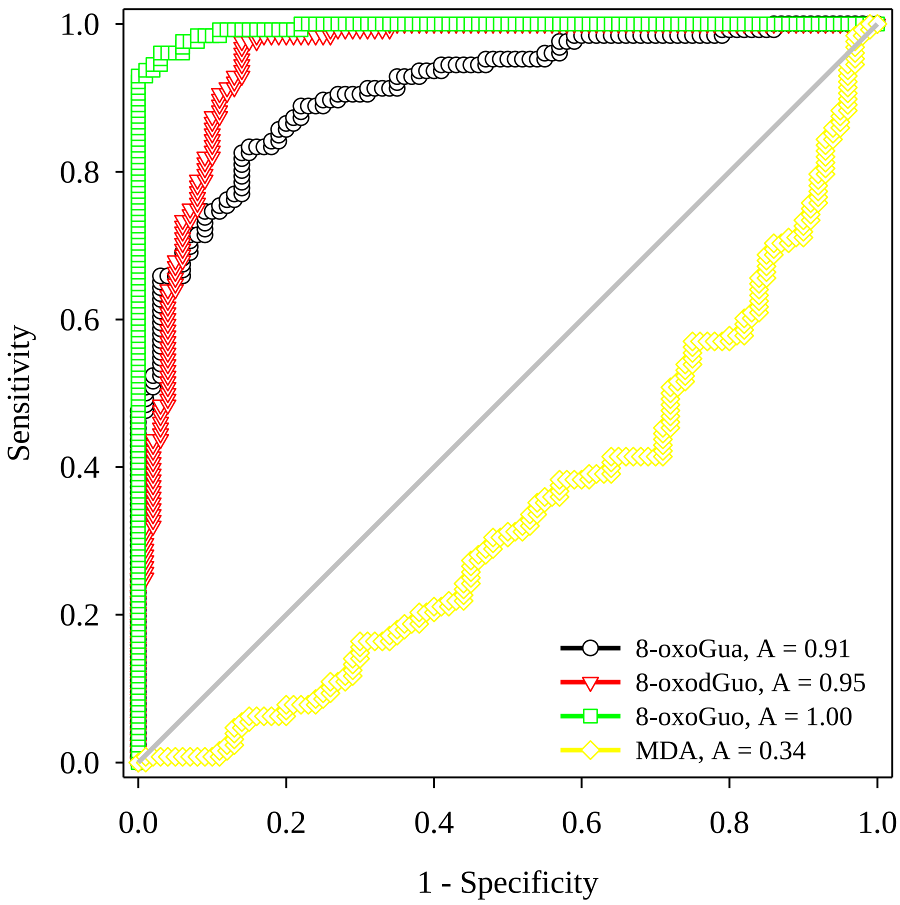

Figure S7. ROC curve analysis of multipale biomarkers for healthy subjects and MV patients (without COPD patients). Urinary biomarkers concentrations were adjusted for creatinine.
